# Supplementary material for: Temporal genomic analysis of melanoma rejection identifies regulators of tumor immune evasion
Source: bioRxiv. 2023 Nov 29:2023.11.29.569032. Preprint. [Version 1] doi: 10.1101/2023.11.29.569032 (PMC10705560; doi:10.1101/2023.11.29.569032)

Supplementary Figure 1- Clinical data and characterization of the Single-cell clones. A) Boxplot of survival times (in days) for low vs. high ITH patients for TCGA melanoma patients (excluding censored data). The individual points denote the survival time for patients in the respective categories. P-value was determined using the Wilcoxon's test between low and high ITH patients, without censoring. **B)** Kaplan-Meier survival curves (time is measured in days on the x-axis) of the cytolytic score of TCGA melanoma patients with low ITH. P-values were determined using the log-rank test to compare the overall survival probability between patients with high (in red) and low (in blue) cytolytic scores. Patients with high cytolytic score have significantly better survival probabilities. **C)** Genetic similarity percentage for each pair of rejected and non-rejected clones. **D)** Evaluation of MHC-I and MHC-II levels in each SCC, measured by flow cytometry. Green color represents the rejected SCCs, pink represents the non-rejected SCCs, and grey represents the parental UVB irradiated cell line. **E)** Somatic mutation profiles of the rejected and non-rejected SCCs. A Venn diagram showing the overlaps of non-silent somatic mutations across the clones, based on whole exome sequencing data. The numbers of non-silent somatic mutations shared and unique to the clones are labeled in the diagram, regions without labels contain no non-silent somatic mutation. The SCC31 (red) and SCC40 (blue) clones on the left-hand side are rejected, the rest are non-rejected.

Supplementary Figure 2- Cell types from scRNA-seq of tumors from mice inoculated with rejected or non-rejected SCCs. A) Heatmap of cluster marker expression among cell type clusters derived from scRNA-seq of rejected and non-rejected SCCs. Color indicates z-score normalized expression per gene. **B)** Boxplot of cell type proportions of macrophage (top) and T cell (bottom) clusters from rejected and non-rejected SCCs at day 6, 10, 16, and 20 after tumor inoculation. Statistical testing by two-sided Wilcoxon test. **C)** UMAP of re-clustered immune cell clusters obtained from scRNA-seq of tumors from mice inoculated with rejected or non-rejected SCCs (left). Bar plots of immune cell type proportions per SCC group at day 6, 10, 16, and 20 after tumor inoculation (right). **D**) Dot plot showing expression of T cell

genes among the T cell clusters presented in (**C**). **E)** UMAP expression plots of *Fcgr3* and *Ki67* among cell-type clusters of the immune compartment. **F)** UMAP of re-clustered myeloid cell clusters obtained from scRNA-seq of tumors from mice inoculated with rejected or non-rejected SCCs (left). Bar plots of myeloid cell type proportions per SCC group at day 6, 10, 16, and 20 after tumor inoculation (right). **G)** Distribution and composition of Mon/Mac subclusters between different time points in rejected (top) and non-rejected (bottom) groups.

Supplementary Figure 3- Differential expression analysis of rejected vs. non-rejected tumor cells. A-B) Differentially expressed genes in the rejected **(A)** vs. non-rejected **(B)** SCCs at different time points post-inoculation.

Supplementary Figure 4- Supplementary data for the *in vivo* CRISPR screen. A) Capture rate of the sgRNA pool among CRISPR-edited SCC35 libraries. Numbers indicate the percentage of sgRNAs that are missing or captured in each library. **B)** Growth curves of SCC35 (n=2) and CRISPR-edited SCC35 (n=3) tumors in immunocompetent mice. **C)** Log fold change of non-targeting control guides and sgRNAs targeting *Cd47* (depletion control) and *Pten* (enrichment control) between day 20 post-inoculation and day 0 input. Enriched and depleted guides are colored with red and blue lines respectively. **D)** Gene-level depletion and enrichment of CRISPR-edited libraries. The score represents the average log2-fold change of guide counts at day 20 post-inoculation and day 0 input libraries. Highlighted in the left square are the top depleted genes ranked by the score.

Supplementary Figure 5- scRNA-seq characterization of *Mif* KO tumor clones. A) Western blot for MIF and Tubulin in SCC35 and SCC37 cells. **B)** *Mif* KO using CRISPR in the non-rejected SCC37 significantly decreased tumor growth compared to the *Mif* WT control clones. Statistical analysis between *Mif* KO to WT expressing clones at day 27 (the latest time point in which all groups are represented) gave a P value of 0.00004 by two-sided Wilcoxon-test followed by Bonferroni correction. n=7-18 **C)** Violin plots of *Mif* expression among tumor cells derived from scRNA-seq of *Mif* KO and control tumors at days 0, 6, 10, and 16 post-inoculation. Statistical testing by two-sided Wilcoxon-test (**** indicates p-value < 0.0001). **D)** Distribution and composition of Mon/Mac subclusters between different time points in *Mif* KO (top) and control (bottom) groups. **E)** UMAP of re-clustered T cell populations from SCC35-derived tumors with or without *Mif* KO. **F)** Heatmap of T cell marker expression among T cell clusters from scRNA-seq of *Mif* KO and control tumors. Color indicates z-score normalized expression per gene. **G)** Longitudinal frequencies of re-clustered CD8 (top) and CD4 (bottom) T cell types from **(E)** in control and *Mif* KO tumors.

Supplementary Figure 1

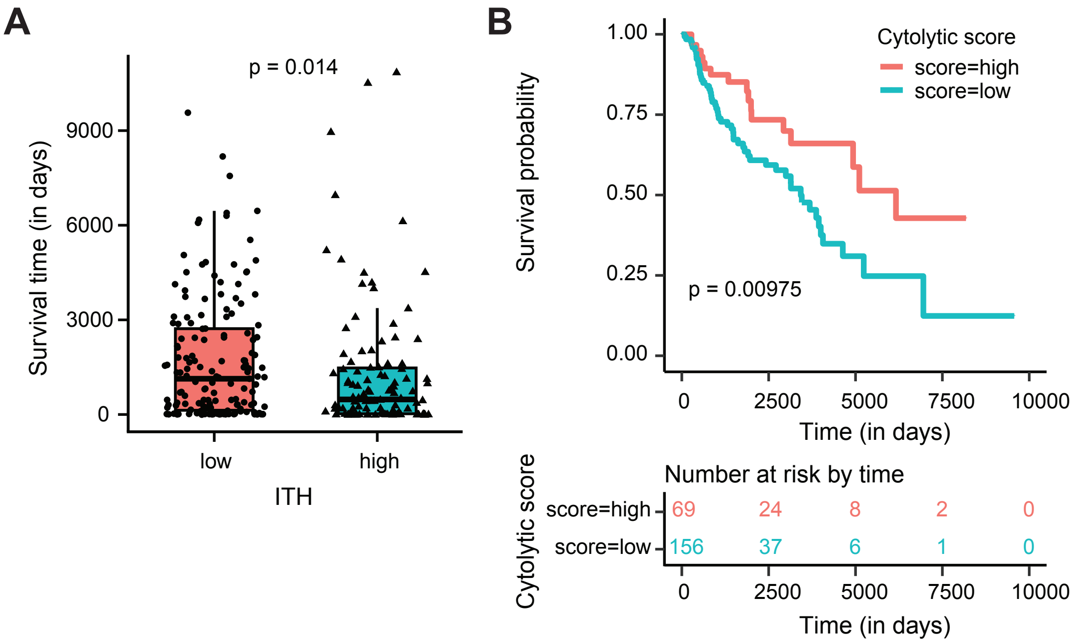

C

Clone numbers		% Similarity
31	32	89.84
31	35	84.75
31	37	83.57
40	32	88.50
40	35	82.45
40	37	81.52

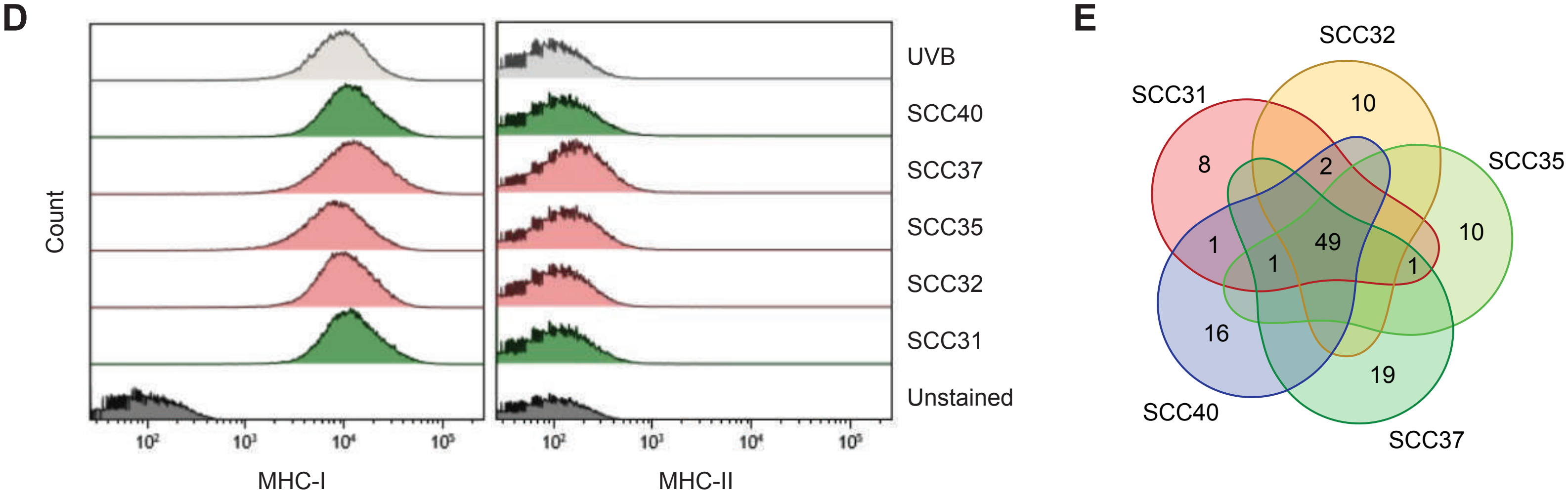

Supplementary Figure 2

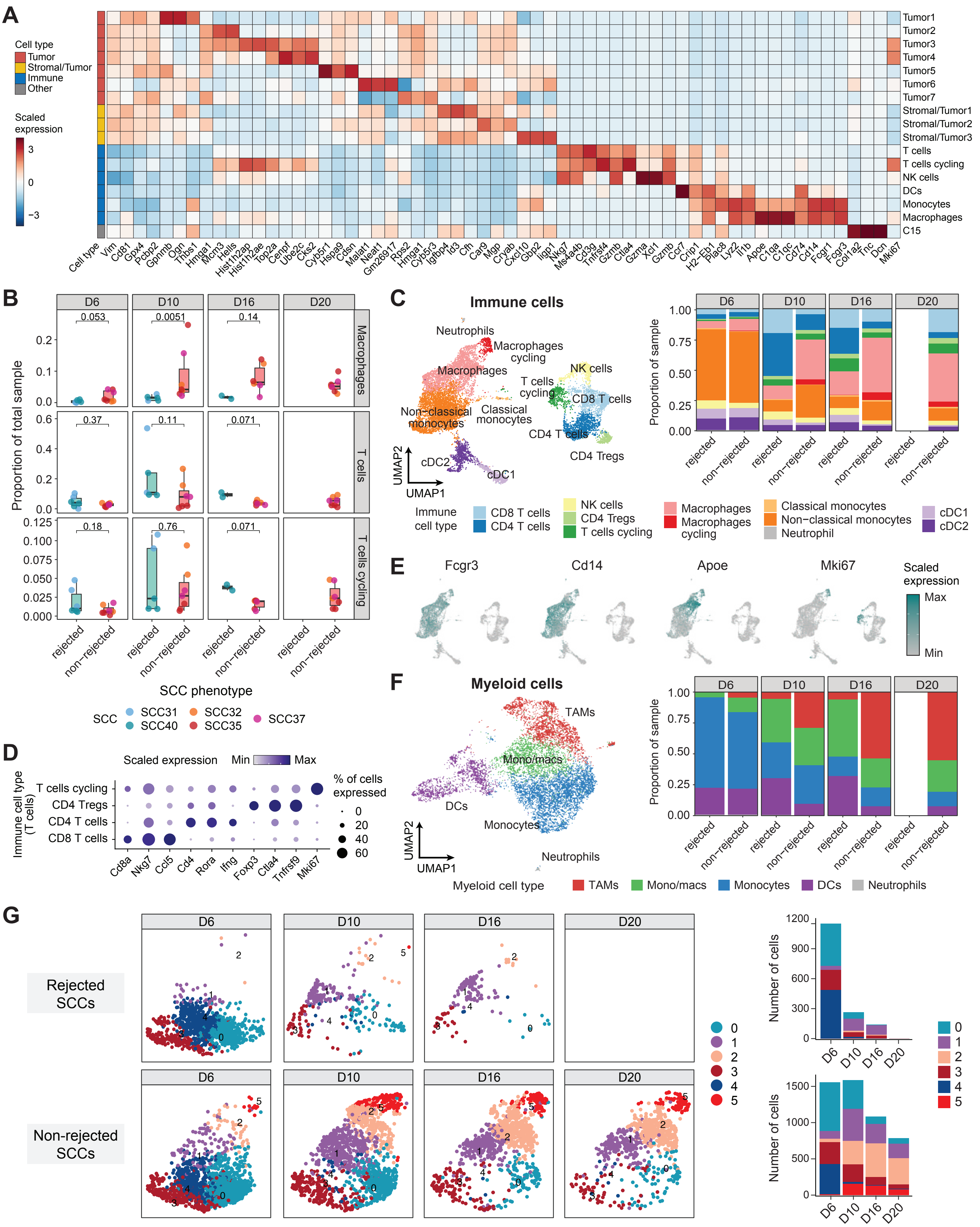

Supplementary Figure 3

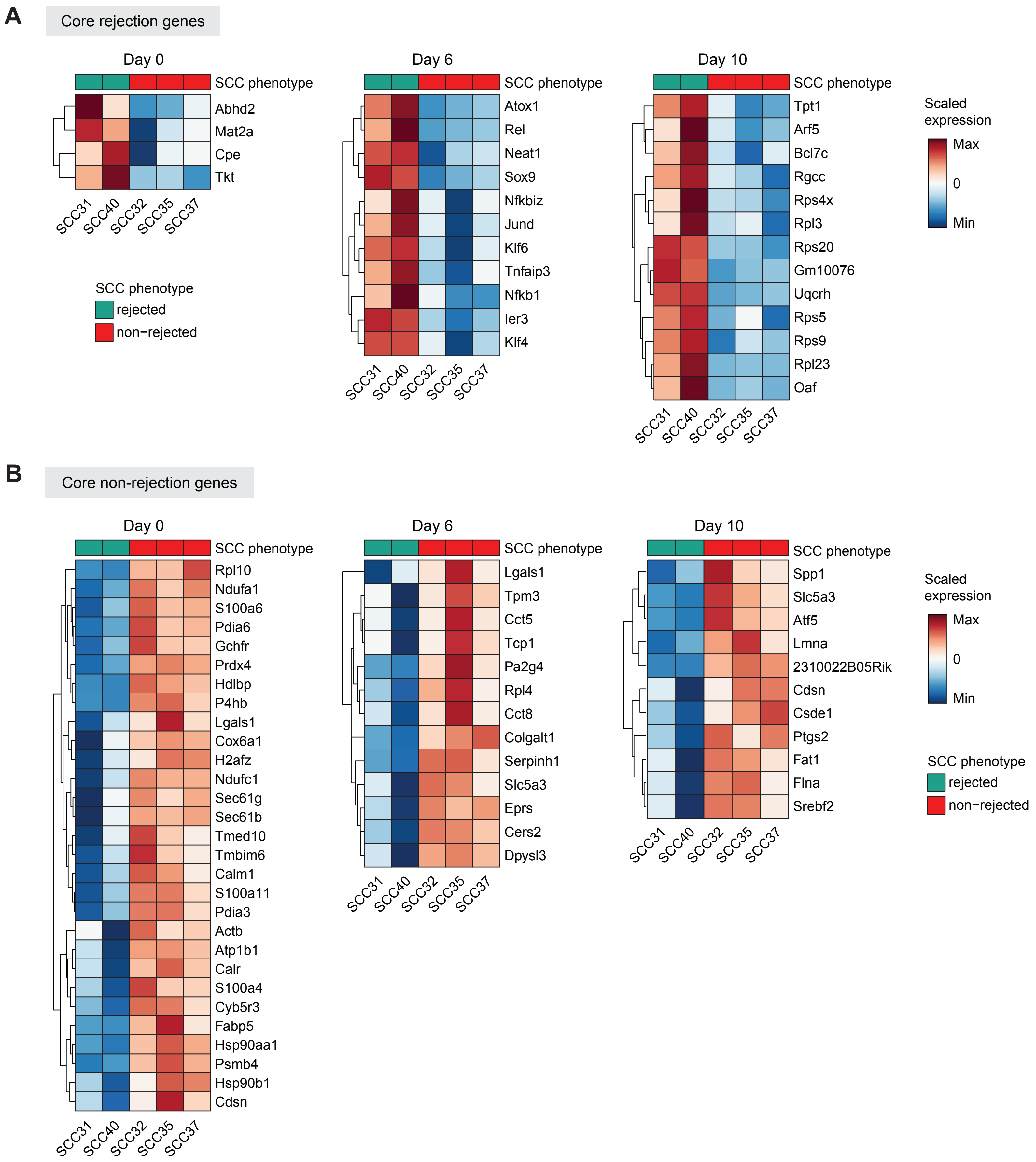

Supplementary Figure 4

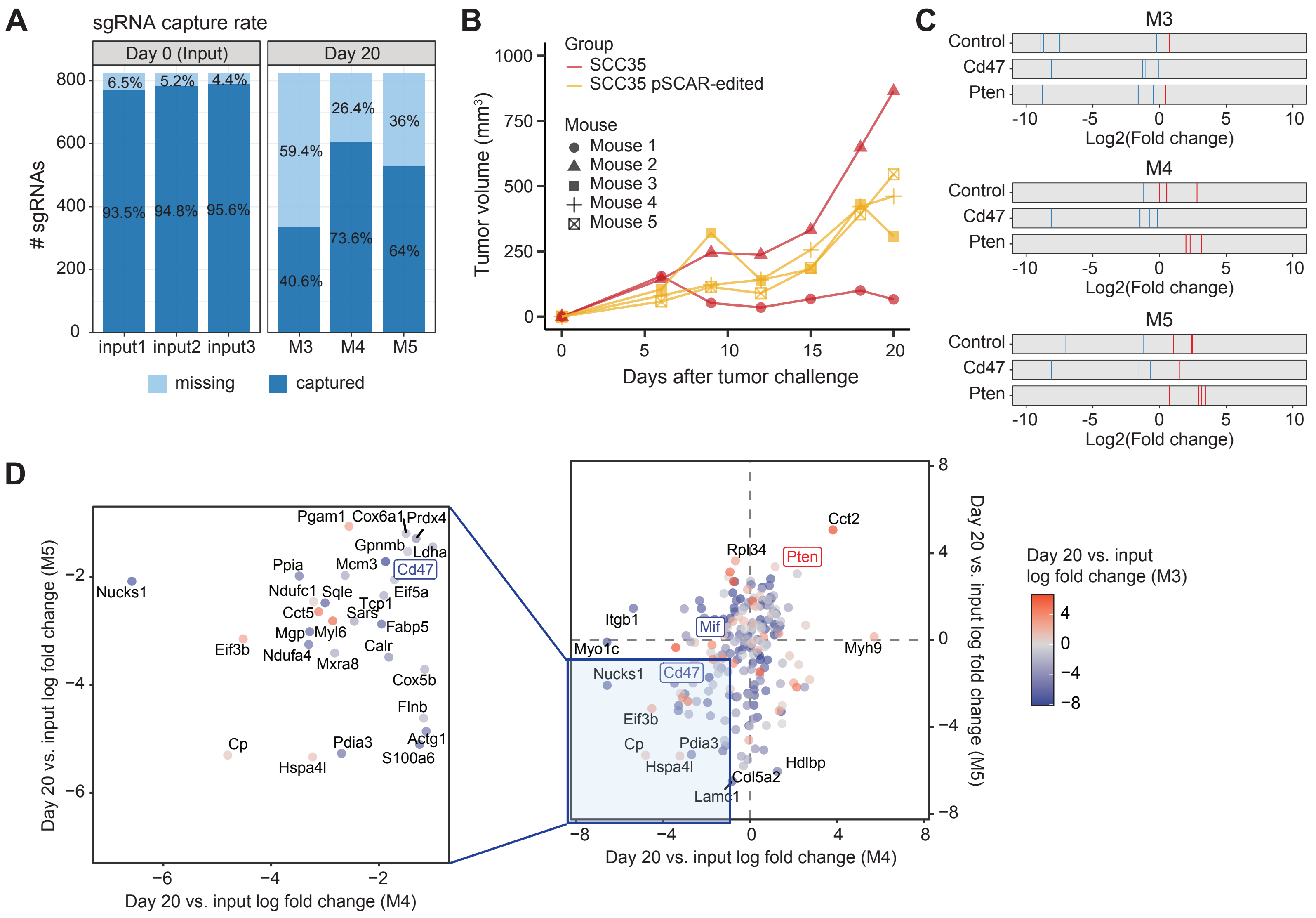

Supplementary Figure 5

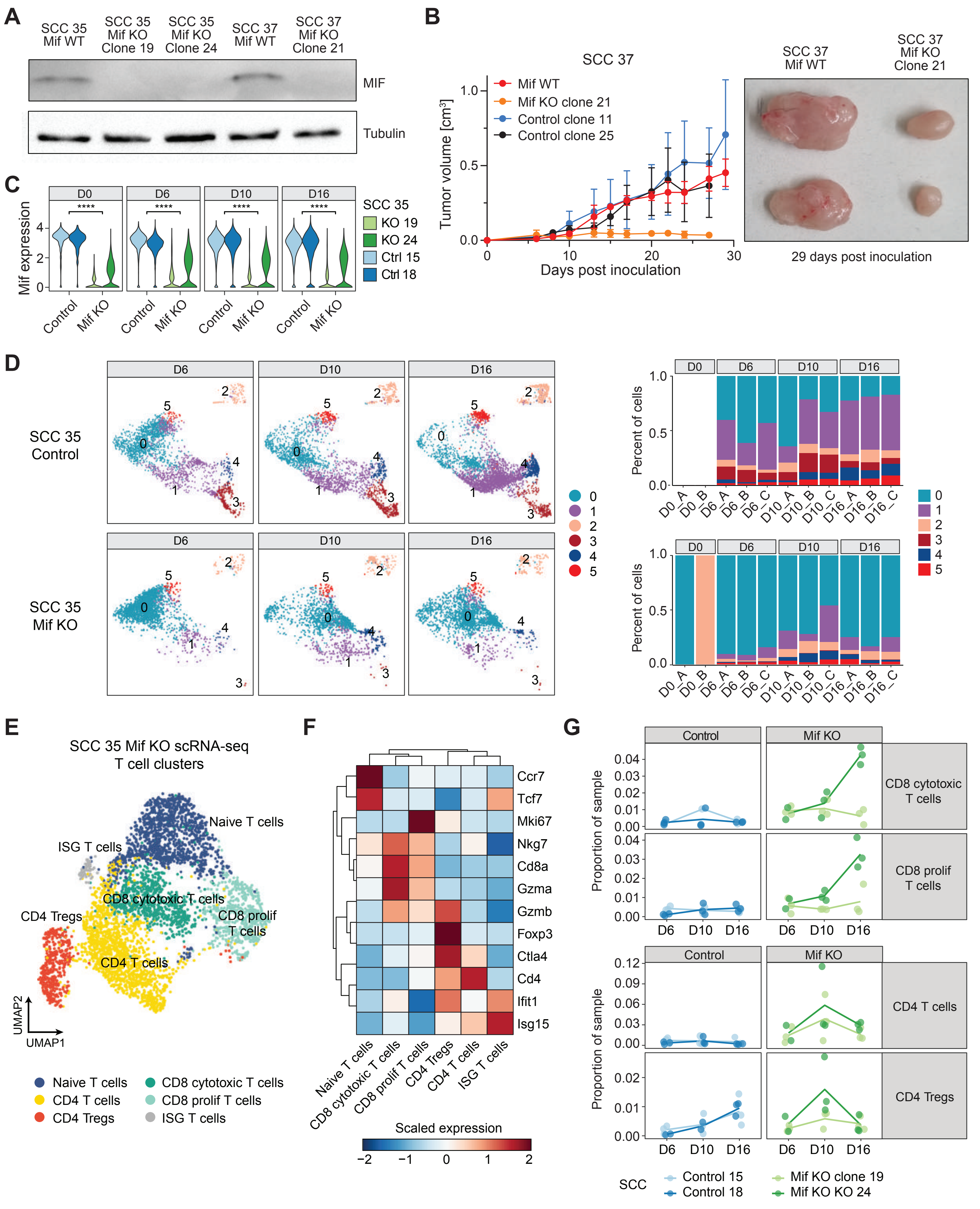

Supplement: 1 [file NIHPP2023.11.29.569032V1-supplement-1.pdf]
